# Reversed oxygen sensing using colloidal quantum wells towards highly emissive photoresponsive varnishes

**DOI:** 10.1038/ncomms7434

**Published:** 2015-03-16

**Authors:** Monica Lorenzon, Sotirios Christodoulou, Gianfranco Vaccaro, Jacopo Pedrini, Francesco Meinardi, Iwan Moreels, Sergio Brovelli

**Affiliations:** 1 Dipartimento di Scienza dei Materiali, Università degli Studi di Milano-Bicocca, via Cozzi 55, I-20125 Milano, Italy; 2 Istituto Italiano di Tecnologia, via Morego 30, IT-16163 Genova, Italy; 3 Department of Physics, University of Genoa, via Dodecaneso 33, 16146 Genova, Italy

## Abstract

Colloidal quantum wells combine the advantages of size-tunable electronic properties with vast reactive surfaces that could allow one to realize highly emissive luminescent-sensing varnishes capable of detecting chemical agents through their reversible emission response, with great potential impact on life sciences, environmental monitoring, defence and aerospace engineering. Here we combine spectroelectrochemical measurements and spectroscopic studies in a controlled atmosphere to demonstrate the ‘reversed oxygen-sensing’ capability of CdSe colloidal quantum wells, that is, the exposure to oxygen reversibly increases their luminescence efficiency. Spectroelectrochemical experiments allow us to directly relate the sensing response to the occupancy of surface states. Magneto-optical measurements demonstrate that, under vacuum, heterostructured CdSe/CdS colloidal quantum wells stabilize in their negative trion state. The high starting emission efficiency provides a possible means to enhance the oxygen sensitivity by partially de-passivating the particle surfaces, thereby enhancing the density of unsaturated sites with a minimal cost in term of luminescence losses.

## Abstract (web summary)

Colloidal quantum wells have great potential as solution-processed light sources. Here, Lorenzon *et al*. demonstrate that such colloidal quantum wells can also be exploited as luminescent-sensing varnishes capable of detecting chemical agents through their reversible emission response.

## Graphical abstract

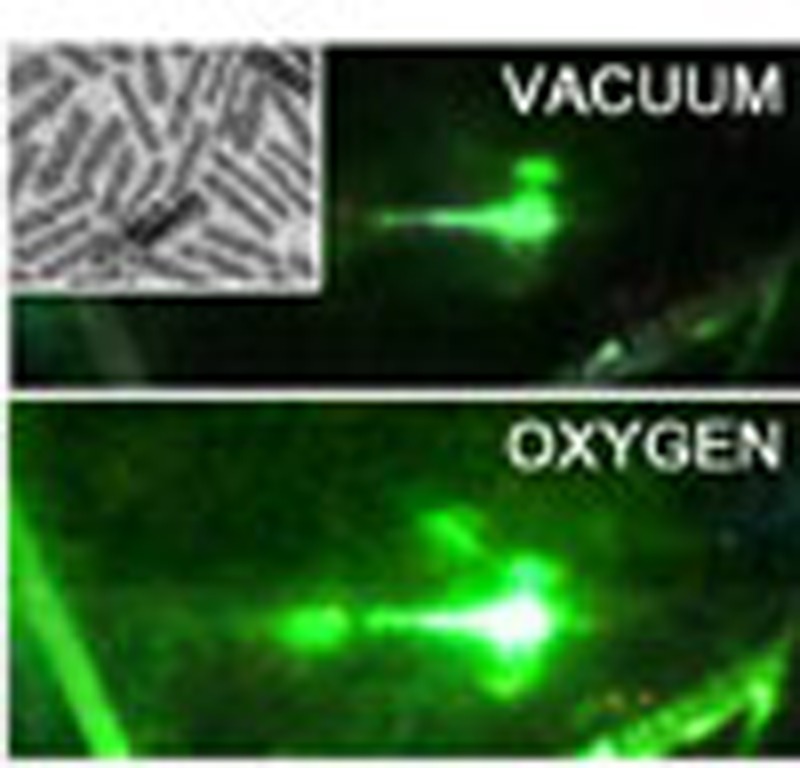

Colloidal semiconductor nanostructures are solution-processable functional materials with great applicative potential in a variety of technologies, ranging from light-emitting diodes^1^, photovoltaic cells^2^ and lasers^3^,^4^ to luminescent markers^5^, plasmonics^6^, single-photon sources^7^, nano-magnetic devices^8^ and luminescent solar concentrators^9^. The particular interest in this class of chemically synthesized systems arises from their tunable emission spectrum and high emission efficiency^10^, as well as an easily manipulated surface chemistry and compatibility with solution-based fabrication processes. Among various types of semiconductor materials, CdSe and CdS have received particular attention over the years, which has lead to the realization of a variety of zero-dimensional structures including spherical core/shell nanocrystals^11^, axial dot-in-rods^12^,^13^ and tetrapods^14^, dot-in-plates^15^ and dot-in-bulk nanocrystals^16^,^17^, as well as one-dimensional (1D) nanorods^14^, nanoribbons^18^ and rod-in-rod structures^19^.

More recently, growing attention is being dedicated to bidimensional nanostructures such as colloidal quantum wells (CQWs)^20^. These strongly anisotropic systems, typically 1–2 nm-thick and tens of nm in lateral dimensions, exhibit 1D confinement of the carrier wave functions resulting in absorption profiles typical of quantum wells^20^,^21^, narrow emission spectra and giant oscillator strength^21^,^22^. Furthermore, CQWs possess exceptionally large exciton^3^,^21^,^23^ and biexciton binding^3^,^23^ energy and suppressed Auger recombination^22^,^24^, which have allowed us to achieve optically pumped lasing in the continuous wave excitation regime^3^. Despite these remarkable optical properties, the CQWs emission efficiency, similar to that of zero- and one-dimensional systems, is typically limited by non-radiative carrier trapping into surface defects that becomes particularly detrimental owing to their exceptionally large surface/volume ratio. Similarly to spherical or elongated CdSe structures, CdSe CQWs surfaces can be passivated with inorganic layers of CdS that feature small lattice mismatch (~4%) and lead to higher emission yields owing to efficient suppression of surface trapping^25^. Recently, CQWs with highly controlled thickness, shape and surface composition have been obtained, such as core/shell^26^,^27^ and core/crown^28^ CdSe/CdS CQWs, where a CdSe nanosheet is, respectively, vertically sandwiched or laterally surrounded by a CdS shell. The growth of a shell with larger bandgap (CdS bulk *E*_g_=2.4 eV) over a CdSe CQW (bulk *E*_g_=1.75 eV) leads to the formation of a quasi-type II junction^29^,^30^, where the electron delocalizes in the Coulomb potential of the hole that remains instead confined to the CdSe core by the high valence band potential barrier, resulting in red-shifted emission and longer radiative lifetimes^25^.

Despite the tremendous advancements in CQW synthesis^20^,^26^, spectroscopy^31^,^32^ and theoretical modelling^20^,^23^, a thorough comprehension of charge trapping and charging is still lacking in the literature and so are reliable guidelines for optimizing their optical performances and environmental stability through suitable surface passivation strategies. Furthermore, understanding the mechanism of surface trapping would allow us to fully exploit the reversible dimming of the photoluminescence (PL) efficiency observed for individual CQWs under vacuum^32^ to realize novel ‘reverse luminescence sensors’ that become brighter in the presence of oxygen^33^, in contrast to conventional sensors that rely on photodarkening to detect electron-withdrawing agents^34^,^35^,^36^,^37^. These active analytical tools are of great interest for a wide range of applications, spanning from life sciences^37^ to pollution monitoring and aerospace engineering^38^ that would strongly benefit from efficient luminescent-sensing varnishes to probe variations of the oxygen level in the atmosphere. The practical realization of this device concept is, however, limited by the lack of dyes that exhibit the necessary balance between high luminescence efficiency, to provide detectable emission in ambient conditions, and good reactivity, to ensure sufficient sensitivity to chemical agents. Reversible photobrightening in wet gasses has been observed in spherical nanocrystals with unpassivated surfaces^39^ that, however, result in low emission quantum yield (~0.4%) and limited detection range (~25%; ref. 40). Importantly, these systems showed reversible luminescence response to the presence/absence of water molecules but no sensitivity to dry oxygen. Colloidal QWs, with their extremely large surface-to-volume ratio and ultrafast radiative lifetimes, offer the ideal combination of high emission yield, vast reactive surfaces and, as we show below, strong sensitivity to O_2_, which make them highly suitable candidates for this technology.

Here we combine spectroelectrochemical (SEC) experiments and spectroscopic measurements in a controlled atmosphere to demonstrate the ability of CQWs to probe oxidative species through brightening of their PL and to thoroughly investigate the roles of trapping and charging on their photophysics. In order to evaluate the effects of heterostructuring in the trapping behaviour, we performed side-by-side experiments on both core-only CdSe and core/shell CdSe/CdS CQWs. O_2_/vacuum cycles demonstrate the reproducibility of the sensing response for both classes of CQWs. Importantly, time-resolved PL experiments in a controlled atmosphere, corroborated by circularly polarized magneto-PL measurements at 2.5 K indicate that, similarly to spherical core/shell nanocrystals, hetero-CQWs stabilize in their negatively charged state under vacuum. Application of an electrochemical potential allows us to recreate the effect of oxidative or reducing environments on the CQW’s PL in a highly controlled fashion and thereby to relate the observed sensing behaviours to changes in the occupancy of surface traps. Remarkably, by lowering the Fermi energy under positive potentials, we observe brightening of the PL of core-only CQWs and strong dimming of their emission when the Fermi level is raised at negative potentials, which confirms the dominant role of hole trapping over electron trapping in the quenching mechanism. In contrast, core/shell CQWs undergo luminescence quenching in both oxidative and reducing conditions. This behaviour is ascribed to dynamic competition between electron and hole trapping resulting from the combined effect of reduced sensitivity of core-localized holes to surface defects and to recovered competitiveness of electron trapping over radiative recombination that, in quasi-type II heterojunctions, is slow due to reduced electron–hole overlap^29^. In order to rationalize the SEC observations in terms of competition between the involved recombination channels, we develop a dynamic model that links the occupancy of hole and electron traps to the emission efficiency for both core-only and core/shell CQWs. Importantly, using core-only CdSe CQWs, we achieve a dynamic sensing range of 90% between atmospheric and 0.5 mbar vacuum condition with a remarkable initial emission quantum yield of ~35%, which demonstrates the suitability of this class of nanostructures for the realization of efficient and sensible luminescent oxygen-sensing varnishes.

## Results

### Synthesis and optical properties of CQWs

**Figure 1:**
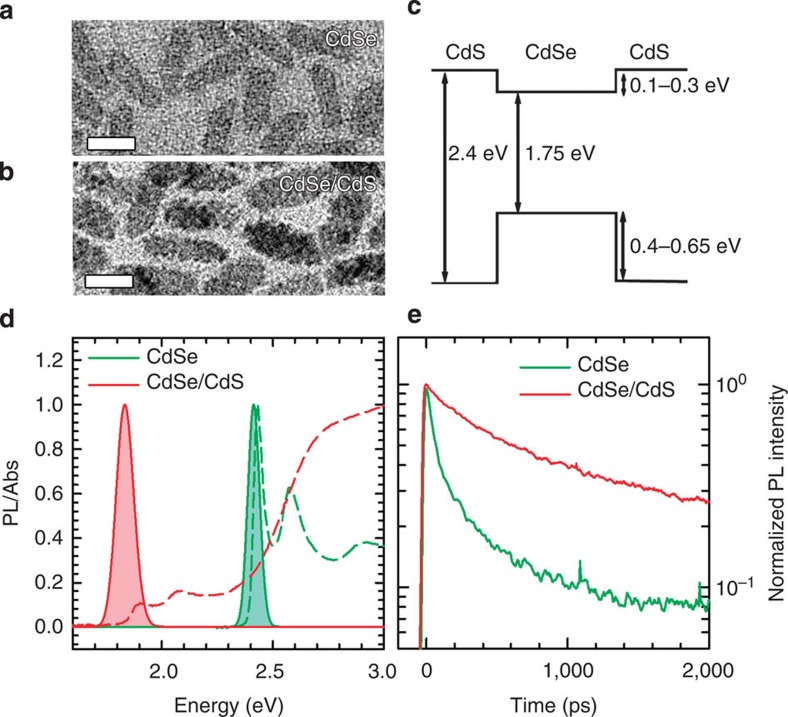
Structure and optical properties of colloidal quantum wells. Transmission electron micrographs of (**a**) core-only CdSe and (**b**) core-shell CdSe/CdS CQWs (shell thickness *H*=0.95 nm corresponding to ~3 CdS monolayers). Scale bars, 25 nm. (**c**) A band alignment diagram of bulk CdSe and CdS. (**d**) Optical absorption (Abs; dashed lines) and normalized PL (solid lines, shaded in colour) spectra of a hexane solution of CdSe (green lines) and CdSe/CdS (red lines) CQWs (excitation at 405 nm). (**e**) Photoluminescence (PL) decay of core-only (green line) and core-shell (red line) CQWs recorded with a streak camera using 3.1 eV excitation.

The CdSe CQWs used in the present study have been synthesized using the synthetic route introduced in ref. 3, which was based on the procedure described in ref. 20. Because of the high surface-to-volume ratio, considerable attention was paid to the purification processes, which was executed without non-solvents such as ethanol or methanol, in order to avoid a reduction of the PL quantum efficiency. The CdSe CQWs are ~2 nm thick, equivalent to ~5 monolayers of CdSe, with average lateral dimensions of 8.8 nm × 36.5 nm (standard deviations of 0.94 and 2.3 nm, respectively) as shown in the transmission electron microscope images in Fig. 1a. To obtain CdSe/CdS core/shell hetero-CQWs, the cores were overcoated on each side with three monolayers of CdS following the layer-by-layer procedure developed by Ithurria and Talapin^26^. The total thickness of core/shell systems is ~4 nm and lateral dimensions are 13.1 nm × 40.7 nm (standard deviation of 1.3 nm × 1.9 nm; Fig. 1b).

The typical band structure of a CdSe core coated with a CdS shell is depicted in Fig. 1c, showing the ~450–650 meV offset between the valence band energies that leads to strong localization of the hole wave function in the core region. In contrast, the smaller 100–300 meV conduction band offset between CdSe and CdS (the exact values are still disputed)^41^ results in the partial delocalization of the electron wave function. Figure 1d reports the optical absorption (dashed lines) and PL (solid lines) of CdSe CQWs and respective core/shell heterostructures under continuous wave excitation at 3.1 eV. The absorption spectrum of core-only CQWs shows the characteristic narrow peaks associated with optical transitions of heavy- and light holes at 2.43 and 2.57 eV, respectively, and to transition from the spin–orbit split-off sub-band to the conduction band at 2.93 eV. The PL spectrum is peaked at 2.41 eV and is Stokes shifted by ~20 meV from the heavy hole absorption feature. Core/shell CdSe/CdS CQWs show broader and red-shifted absorption and emission spectra, as expected from reduced quantum confinement resulting from partial delocalization of the electron wave function over the shell region^24^,^25^. The PL decay curves are shown in Fig. 1e. In agreement with previous observations, CQWs ensembles as well as individual particles^32^ exhibit sub-nanosecond PL lifetimes^21^ and non-exponential decay dynamics at room temperature. Similar to spherical core/shell nanocrystals^41^ and axial CdSe/CdS heterostructures^12^, reduced overlap between electron and hole wave function leads to an extended radiative PL lifetime for hetero-CQWs that, nevertheless, exhibit higher PL quantum yield (Φ_PL_) with respect to core-only CQWs (Φ_PL_~60% versus Φ_PL_~35% in solid film), thanks to reduced charge trapping in surface defects^25^.

### Oxygen-sensing experiments

**Figure 2:**
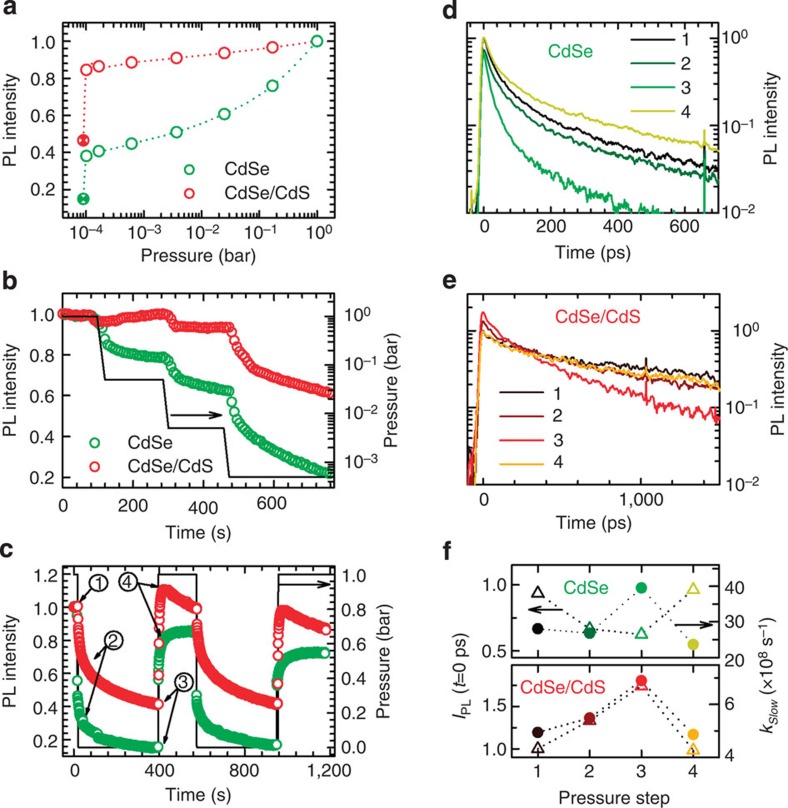
Reversed O_2_ sensing using colloidal quantum wells. (**a**) Integrated Photoluminescence (PL) intensity of core-only (green circles) and core-shell (red circles) CdSe/CdS CQWs as a function of the chamber pressure (logarithmic scale). (**b**) Integrated PL intensity of CdSe (green) and CdSe/CdS (red) CQWs during a stepwise pressure scan. The pressure (black line, logarithmic scale) is reduced rapidly and then maintained constant for ~200 s while the PL transient is simultaneously monitored. (**c**) Integrated PL intensity of CdSe (green) and CdSe/CdS (red) CQWs during ‘ON/OFF’ O_2_/vacuum cycles starting from atmospheric pressure (1 bar) down to 10^−4^ bar. The pressure during the scan is shown as a black line. PL decay curves of (**d**) core-only and (**e**) core/shell CQWs measured at the respective steps of the O_2_/vacuum cycles in ‘**c**’ (highlighted with sequential numbers). (**f**) Decay rate of the slow component of the biexponential dynamics (triangles) and initial PL intensity, *I*_PL_(*t*=0 ps; triangles) for both core-only and core/shell CQWs. The same trends are observed for the fast decay contribution (Supplementary Fig. 11). All measurements are performed at room temperature using 3.1 eV excitation.

To investigate the effect of oxidative environments on the luminescence properties of CQWs and to demonstrate their reversed oxygen-sensing capability, we monitored the evolution of the PL upon lowering the O_2_ pressure in the sample chamber from atmospheric pressure to 10^−4^ bar (Fig. 2a). Interestingly, both systems undergo significant PL quenching with decreasing pressure, thus confirming the surface passivating role of oxygen, whose removal progressively activates surface-quenching sites. The nature of the defects responsible for quenching is provided by the comparison between core-only and core/shell materials, the first showing a much stronger dimming (~90%) than the latter (~55% with respect to the initial value in O_2_). In both core/shell and core-only CQWs, the electron wave function is expected to explore the surfaces essentially equally, given the ca. 1 nm thickness of the CdS shell (3 monolayers of CdS; Fig. 1b). Therefore, electron trapping alone cannot be held accountable for the observed different sensitivity to surface chemistry. The strong difference between the two samples lies instead in the accessibility of surface traps for core-localized holes, which is drastically reduced in CdSe/CdS CQWs. The observed stronger quenching for core-only materials, therefore, points to a key role of hole trapping in the quenching mechanism and outlines the ability of oxygen to passivate excess electrons on the CQW’s surfaces. This picture is further confirmed by oxygen-sensing measurements on core/shell CQWs with thinner shell (*h*=0.32 nm, corresponding to ~1 monolayer of CdS) that shows intermediate behaviour (~65% PL quenching) between core-only and core/shell CQWs with *h*=0.95 nm (Supplementary Fig. 1). Importantly, no shift of the PL spectrum of both core-only and core/shell CQWs is observed during the pressure ramp (Supplementary Fig. 2), which indicates that the observed trends are due to activation/passivation of surface traps and not to oxidation/reduction of the CQW surfaces, as instead observed for spherical nanocrystals exposed to humid air^35^.

Interestingly, we notice that the PL drop occurs in two distinct time ranges, one instantaneous with the O_2_ pressure change, leading to ~60 and ~15% dimming for core-only and core/shell CQWs, respectively, followed by a slow gradual decrease in efficiency at constant pressure (~10^−4^ bar), which accounts for the residual PL loss (semi-filled symbols in Fig. 2a). To better understand this effect, we monitored the PL of both CQWs during stepwise pressure ramps. The data reported in Fig. 2b show similar phenomenology for core-only and core/shell CQWs (although more pronounced for core-only systems for the reasons explained above): at low vacuum levels (*P*~50−5 mbar), the PL intensity correlates instantaneously with the chamber pressure, with an initial rapid drop concomitant to evacuation, followed by a plateau at constant pressure. On the other hand, at higher vacuum levels (*P*<0.5 mbar), the dimming is more pronounced and proceeds in time even after the chamber pressure has reached full saturation. These observations point to the coexistence of two quenching regimes that reciprocally dominate the sensing behaviour in different vacuum conditions. The first is responsible for the stepwise response to the chamber pressure and is ascribed to the rapid extraction of O_2_ molecules weakly bound to the CQW film that passivate surface sites. The second leads instead to the slow progressive dimming in high vacuum conditions and is most likely owing to the gradual desorption of adsorbates that require a larger driving force to detach from the CQWs surfaces.

This interpretation is supported by the ON/OFF pressure cycles reported in Fig. 2c that also demonstrate the reproducibility of the sensing response. In these experiments, the sample chamber was rapidly evacuated from atmospheric pressure (1 bar in O_2_) to ~10^−4^ bar, while simultaneously monitoring the PL over time. Once the PL intensity had reached a plateau, the sample chamber was instantaneously filled with O_2_, so as to re-establish the initial pressure. Also, in this case, the PL was monitored until saturation before starting the successive cycle. Once again, we observe the rapid quenching for both core-only and core/shell CQWs in the initial few seconds of each cycle, concurrently of the removal of ~99.99% of O_2_ from the sample chamber. Successively, the PL quenching proceeds slower while the pressure remains unchanged. Interestingly, pumping O_2_ back in the chamber results in a symmetric trend: initially, the PL brightens rapidly, owing to largely increased availability of gaseous molecules, and proceeds slowly at atmospheric pressure as residual reactive defects are gradually saturated. The same behaviour is observed for several consecutive cycles confirming the reproducibility of the sensing response and ensuring that the capping ligands are preserved. Detailed characterization of the sensing response including extended scans of over 10 consecutive cycles, batch-to-batch reproducibility and stability tests are reported in Supplementary Figs 3–5, highlighting the reproducibility of the luminescence-sensing response. It is worth noting that refilling the sample chamber with nitrogen leads to no recovery of the PL intensity, which further confirms the essential role of oxygen, and not of the pressure itself, in passivating trapping sites. To test the sensitivity of CQWs to other gaseous species, we performed ON/OFF pressure cycles using both carbon dioxide and carbon monoxide/argon mixture (Supplementary Fig. 6). CQWs show reversible photobrightening also in the presence of CO and CO_2_ although to a lesser degree with respect to oxygen, which might further extend their potential use in environmental gas sensors. Importantly, the sensing response is reproducible also in the presence of humidity (Supplementary Fig. 7), thus making these systems particularly suitable for gas flow sensing in ambient conditions.

### Time-resolved spectroscopy of CQWs

In order to gain deeper insight into the quenching mechanism in different pressure conditions, we measured the PL decay dynamics of both samples in key steps of an ON/OFF pressure cycle as indicated by numbers in Fig. 2c, namely, step 1, the initial condition of atmospheric pressure; step 2, the stage at which the chamber pressure is decreasing to ~10^−4^ bar; step 3, the end of the asymptotic PL dimming and step 4, the maximum of the PL signal after recovery. The PL decay curves of core-only and core/shell CQWs are reported in Fig. 2d,e, respectively. The complete set of time-resolved PL data is shown in Supplementary Figs 8 and 9. In any O_2_ pressure condition, both systems show double exponential decay curves with a fast initial portion followed by a slower decay (respective lifetimes *τ*_FAST_ and *τ*_SLOW_) that accounts for ~70% of the total luminescence of CdSe CQWs and for over 90% of the CdSe/CdS CQWs emission (the curve fits and fitting parameters are reported in Supplementary Table 1 and Supplementary Fig. 10). This suggests that both ensembles consist mainly of two subpopulations of CQWs, one decaying primarily radiatively and the other one being dominated by non-radiative exciton relaxation. Specifically, we ascribe the long-lived component to radiative recombination of the subpopulation of CQWs with suppressed surface trapping, which is larger in the CdSe/CdS CQW sample, thanks to the passivation effect of the wide bandgap shell, as also confirmed by the higher PL quantum yield with respect to the core-only material (60% versus 35%). On the other hand, the fast decay portion is assigned to trap-assisted recombination of the fraction of CQWs whose surface defects are not fully passivated by either the organic ligands or by O_2_, and thus provide an additional efficient non-radiative recombination channel. In Fig. 2f, we report the decay rate of the long-lived emission that accounts for the majority of the signal (*k*_SLOW_=1/*τ*_SLOW_) at the various stages of the pressure ramp of Fig. 2c, together with the zero-delay PL intensity values 
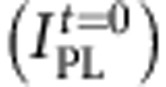
, indicating the initial excited-state population for both material systems. Although the quantitative description of the luminescence response is beyond the scope of this work, monitoring the evolution of these two parameters in the absence/presence of a specific analyte is instructive to distinguish between so-called ‘static’ and ‘dynamic’ quenching mechanisms^42^. Specifically, a quenching process is typically considered as ‘static’ when it occurs on a timescale significantly faster than radiative recombination and thus lowers 
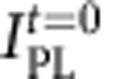
 without modifying the decay kinetics. In contrast, a dynamic quenching process takes place concurrently to emission, resulting in accelerated decay rate, while 
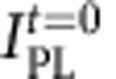
 remains largely constant^42^.

With this in mind, we first examine the behaviour of core-only CdSe CQWs. Upon evacuating the sample chamber from atmospheric pressure to 10^−4^ bar (step 2), we observe an ~30% reduction of 
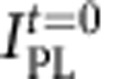
 while *k*_SLOW_ is essentially unaffected (Fig. 2f). In direct opposition, at step 3, the decay rate almost doubles its value while 
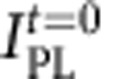
 remains unchanged. The same trend is observed for the fast radiative rate, *k*_FAST_, as reported in Supplementary Fig. 11. This behaviour supports the picture of the following two coexisting quenching processes: a static quenching mechanism that dominates at higher pressures and extracts photogenerated carriers on a timescale faster than radiative decay^42^, and a slow dynamic quenching regime where gas desorption activates less efficient surface traps, and thereby modifies the PL kinetics without affecting the initial excited-state population. Finally, upon pumping O_2_ back in the sample chamber, the initial decay profile is re-established (point 4). Single-particle PL-sensing measurements performed for a set of 30 CQWs confirm the distribution of the sensing response throughout the ensemble (Supplementary Fig. 12). Specifically, ~20% of the investigated CQWs show strong quenching (≥90% of the initial PL intensity), 17% undergo weak dimming of their emission efficiency (≤40%), while the remaining 60% show intermediate sensitivity. Importantly, the sensing response is independent on the initial PL intensity, as highlighted in the correlation plot of the PL quenching versus initial PL intensity reported in Supplementary Fig. 12.

Analysis of the time-resolved PL measurements reveals a fundamentally different behaviour for heterostructured CQWs with respect to core-only materials (Fig. 2e,f). Upon proceeding from steps 1 to 2 to 3 in the pressure ramp, the decay rate gradually grows, accompanied by an increase of 
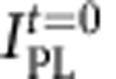
, which implies that more photons are initially emitted at reduced pressure. Concomitantly, however, the PL quantum yield drops (Fig. 2c), indicating that the effect cannot be due to suppressed ultrafast trapping. These spectroscopic signatures are instead consistent with the recombination of charged excitons (trions), bound states composed of two carriers Coulombically coupled to the same carrier of the opposing sign. In zero-dimensional quantum dots, this typically results in doubled radiative decay rate and larger initial PL intensity with respect to neutral excitons^43^,^44^. Negative trion emission under vacuum has been recently observed in single-particle experiments of so-called giant core/shell CdSe/CdS nanocrystals, which show blinking-free trion emission with ~100% quantum yield at room temperature^45^ and circularly polarized PL under magnetic fields at 4 K, as a result of unbalanced spin population of the Zeeman-split trion sublevels^46^. In our case, efficient trion emission in vacuum could partially compensate for the activation of surface traps and thus contribute to the reduced PL sensitivity to the chemical environment of hetero-CQWs with respect to core-only materials.

### Magneto-optical properties of CQWs

**Figure 3:**
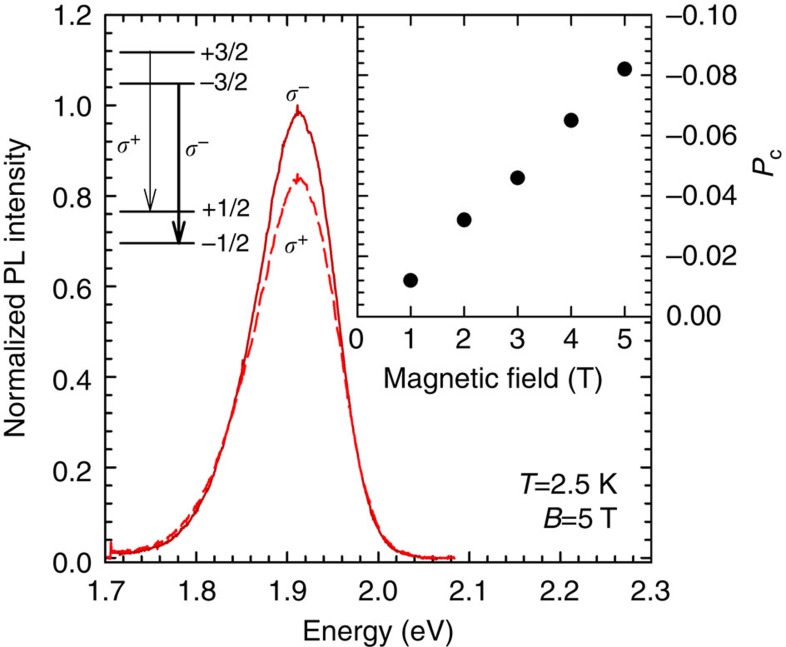
Magneto optics of negatively charged core/shell CQWs. Circular polarization-resolved Photoluminescence (PL) spectra of CdSe/CdS CQWs at magnetic field of *B*=5 T and *T*=2.5 K measured using 3.1 eV excitation. The clockwise (*σ*^+^) and anticlockwise (*σ*^−^) emissions are reported in dashed and solid lines, respectively. Inset: magnetic field dependence of circular polarization degree (*P*_c_) and scheme of the spin structure and optical transitions for negative trions in external magnetic fields.

In order to unambiguously ascribe the behaviour of core/shell CQWs to trion emission and to investigate whether under vacuum our systems charge with an excess electron, like thick-shell hetero-nanocrystals, we performed circular polarization-resolved PL experiments at cryogenic temperatures under high magnetic fields (Fig. 3). In these experiments, the sample is mounted in the variable temperature insert of a split-coil cryo-magnet with direct optical access and the circularly polarized PL is selected using a quarter-wave plate coupled to a linear polarizer. The degree of circular polarization (*P*_c_) is defined as: *P*_c_=[*σ*^*+*^−*σ*^−^]/[*σ*^*+*^+*σ*^*−*^], where *σ*^*+*^ and *σ*^*−*^ are the intensities of the right-handed (clockwise) and left-handed (anticlockwise) circular polarized emission spectra. The sign of *P*_c_ is directly determined by the sign of the excess charge: a negative *P*_c_ corresponds to CQWs charged with an excess electron, whereas a positive value is observed for the decay of positive trions^46^. The polarized emission spectra of CdSe/CdS CQWs at 2.5 K are shown in Fig. 3 together with *P*_c_ as a function of the magnetic field. Similarly to giant core/shell nanocrystals, core/shell CQWs exhibit negative *P*_c_ that, together with the PL dynamics in Fig. 2e, indicate that under vacuum they are in a negatively charged state. On the other hand, the same measurements performed on core-only CdSe CQWs show no detectable circular polarization, which indicates that the ensemble is in neutral state (Supplementary Fig. 13).

### Spectroelectrochemistry experiments

**Figure 4:**
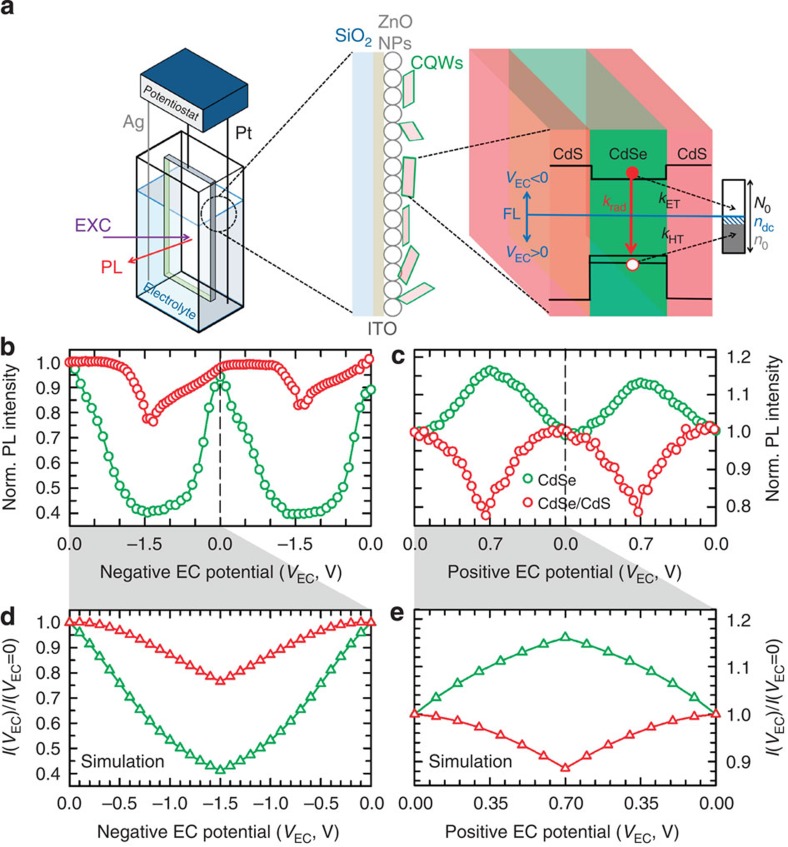
Spectroelectrochemistry measurements on colloidal quantum wells. (**a**) Schematics of the SEC setup: The electrochemical cell with 0.1 M tetrabutylammonium perchlorate in propylene carbonate as an electrolyte and a working electrode comprising an ITO-coated glass covered with a layer of ZnO nanoparticles (NPs) and CQWs on the top of the structure. The excitation (EXC) beam and the emitted Photoluminescence (PL) are indicated as purple and red arrows, respectively. A diagram illustrating the model used to describe the effect of the electrochemical potential on the PL intensity via filling/emptying of trap bands at the surface of the CQWs in response to changes in the position of the Fermi level (FL; blue line). The defect band at the CQW surface is shown on the right of the CQW. (**b**) Normalized (Norm.) spectrally integrated PL intensity of CdSe CQWs (green circles) and core-shell (red circles) CdSe/CdS CQWs during a stepwise scan of the electrochemical (EC) potential to negative values (*V*_EC_<0). (**c**) Stepwise voltage scan for a positive electrochemical potential (from *V*_EC_=0 to +0.7 V). Two potential cycles are reported to show the repeatability of the process. The calculated PL intensity as a function of electrochemical potential using parameters listed in Supplementary Table 1 for core-only CdSe CQWs (green curve) and core/shell CdSe/CdS CQWs (red curve). (**d**) Filling the defect band with electrons (enhancing FL) under negative potential suppresses electron trapping but activates hole trapping leading to PL quenching of both core-only and core/shell CQWs. (**e**) Depleting electrons from the defect band under positive potential enhances electron trapping but suppresses hole trapping resulting in enhanced PL from CdSe CQWs, whose dominant non-radiative channel is hole capture (capture rate *k*_HT_). In contrast, core/shell CdSe/CdS CQWs undergo dimming of the PL efficiency due to activated electron trapping (*k*_ET_).

To further confirm our original assessment of the central role of hole trapping in the photophysics of CQWs and to relate the observed quenching trends to the population of surface defects, we conducted SEC measurements. SEC has been recently used by several groups for studying the mechanisms of luminescence blinking^43^,^47^,^48^, charging^49^ and nanocrystal doping^50^,^51^, as well as to demonstrate the ratiometric sensing capability of multicolour emitting heterostructures^52^. To date, no SEC study of bidimensional colloidal quantum structures is available in the literature. The custom experimental setup used for these experiments is depicted in Fig. 4a. We start by recreating the effects of the removal of oxygen studied in the pressure-controlled experiments by applying a negative electrochemical (EC) potential. In this condition, corresponding to raising the Fermi energy in the CQWs, surface defects are gradually filled with electrons, which activates their hole-trapping capability. Simultaneously, intragap electron traps are progressively passivated. The PL intensity is thus determined by the competition between the quenching effect of hole withdrawal and the brightening effect of suppressed electron trapping. In Fig. 4b, we monitor the evolution of the PL of both core-only and core/shell CQWs in a cyclic stepwise scan from *V*_EC_=0 V to *V*_EC_= −1.5 V and back to 0 V. The full set of PL spectra as a function of negative electrochemical potential is reported in Supplementary Fig. 14. In agreement with the pressure ramps in Fig. 2, upon increasing the negative potential, we observe strong PL quenching for core-only CQWs (~60% reduction), while the core/shell analogues are only weakly affected (20%). Interestingly, these reductions correspond to the PL drops observed during the initial step (steps 1–2) of the pressure ramp in Fig. 2c, indicating that the electrochemical potential affects primarily the surface sites that respond instantaneously to the variation of the chamber pressure.

Next, we analyse the effect of a positive EC potential, which corresponds to lowering the Fermi energy in the CQWs (Fig. 4c and Supplementary Fig. 15). This allows us to artificially expose our materials to an excess of electron-poor agents with respect to the atmospheric condition and thereby to monitor the PL response to an even more severe oxidative environment. The data show a remarkable anticorrelation between the PL response of core-only and core/shell CQWs, with the first undergoing a progressive brightening of the PL intensity (~20% increase) while the latter are concomitantly quenched by a comparable degree. Once again, the increase of PL quantum yield of core-only CQWs under oxidative electrochemical potential can be explained in terms of suppressed hole trapping that dominates over the simultaneous activation of electron traps. The opposite effect describes instead the trend of core/shell CQWs, for which activated electron trapping is the dominant effect, similar to recent observations for CdSe/CdS dot-in-bulk nanocrystals^52^. Taken together, these observations confirm our original assessment that non-radiative recombination in core-only CQWs is dominated by trapping of holes that is suppressed in oxidative environments while core/shell systems are mostly affected by electron traps. In both polarities, when returning back to zero potential, we observe reversible evolution of the two PL signals to their original intensity, indicating that potential sweeps do not cause any permanent chemical degradation of the CQWs. Similar to that observed in the pressure scans, the normalized PL spectra (Supplementary Figs 14 and 15) show no measurable shift under neither positive nor negative EC potential, which confirms that the PL intensity trends are due to changes in the occupancy of surface sites and not to oxidation/reduction of the CQWs.

### Kinetic model of surface trapping in CQWs

To rationalize the SEC data, we propose a kinetic model that links the PL efficiency to the occupancy of surface traps that can be activated/deactivated by raising or lowering the electrochemical potential. The scheme of the photophysical processes occurring in the sample during the SEC measurements is depicted in Fig. 4a. Upon photoexcitation, electron–hole pairs can recombine radiatively with rate *k*_rad_ or non-radiatively in surface defects. To account for the electron and hole traps, we introduce an electron-trapping channel for electrons with rate *k*_ET_, and a hole-trapping channel with rate *k*_HT_. We further assume that electron- and hole-trap sites are continuously distributed in energy across the CQW ensemble forming a ‘trap band’ and only unoccupied sites can trap electrons while only occupied sites can trap holes. At zero EC potential, we consider the Fermi level to be at the centre of the energy gap of the CdSe core, which defines the initial occupancy of trap bands (Fig. 4a). The application of a negative (positive) EC potential raises (lowers) the Fermi energy determining a variation of the trap occupancy. On the basis of these assumptions, we formulate a set of four rate equations for the population of photoexcited charges (conduction band electrons, *n*, valence band holes, *p*) and trapped carriers (*n*_T_ and *p*_T_, for trapped electrons and holes, respectively), and compute the evolution of the PL under oxidative or reductive potentials for a set of experimental parameters that are typical of samples studied in the present work (see page no. 17 in the Supplementary Information and ref. 53 for details and model parameters). The results of the simulation under negative and positive electrochemical potentials are reported in Fig. 4d,e, respectively. This simple model reproduces the main experimental trends with core-only CdSe CQWs undergoing a progressive brightening at increasing positive potential as a result of suppressed hole trapping, whose rate strongly outcompetes electron trapping (*k*_HT_>>*k*_ET_) in agreement with the time-resolved data in Fig. 2d. The same argument explains the trend for negative potentials, where filling empty hole traps with electrons markedly quenches the PL despite the fact that electron withdrawal is concomitantly reduced. Core/shell CQWs exhibit slower PL decay with respect to core-only systems (*k*_rad_~4 × 10^8^s^−1^ versus *k*_rad_~3 × 10^9^ s^−1^ for core/shell and core-only, respectively; Supplementary Table 1). This results in electron trapping becoming competitive with radiative decay. Simultaneously, the core-shell motif suppresses hole trapping and thereby leads to overall increased resilience of the emission yield to the electrochemical environment.

### Demonstration of CQW-based ‘reverse-sensing’ varnishes

**Figure 5:**
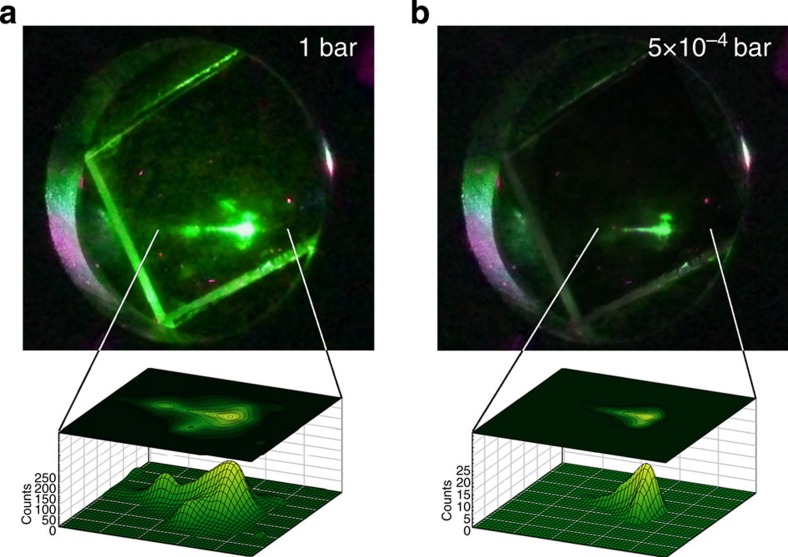
CQWs for reverse oxygen-sensing luminescent varnishes. Photographs and 3D surface intensity plots of a film of CdSe CQWs at (**a**) 1 bar O_2_ pressure and (**b**) 5 × 10^−4^ bar vacuum under 3.1 eV excitation, showing the about one order of magnitude stronger luminescence in atmospheric conditions with respect to vacuum.

The whole body of experimental results presented above indicates that colloidal CQWs are suitable materials for application in oxygen-sensitive coatings able to detect the presence of O_2_ through reversible brightening of their PL. To provide a final proof of their potential, in Fig. 5, we report the photographs of a CdSe CQWs film under ultraviolet illumination in O_2_ and in vacuum, once again highlighting the remarkable difference between the emission intensity in the two conditions. We stress that such sensing range is achieved using an efficient emitter with over 30% PL quantum yield in film form and for pressure variations around atmospheric values. This makes these novel 2D colloidal structures particularly suitable for application as luminescent air-sensing varnishes for applications including air flow studies in aerodynamic research (wind tunnel model) that currently rely on smoke, viscous fluids or evaporating suspensions for revealing transitions from laminar to turbulent flow, as well as flow separation on airplane or car body parts. Furthermore, their high starting emission efficiency provides a possible means to further enhance the environmental sensitivity by partially de-passivating the surfaces of the CQWs and thereby to enhance the density of available surface sites with a minimal cost in term of luminescence losses.

## Discussion

In conclusion, we investigated the processes of charging and trapping in CQWs by means of SEC methods and time-resolved spectroscopy in a controlled atmosphere. The data demonstrates the brightening effect of oxygen on the emission of CQWs whose efficiency is mainly determined by hole trapping in surface defects. SEC experiments reproduce well the observed environmental effects and further confirm the improved optical stability of heterostructured CQWs. The O_2_-sensing process is reversible and could be used to realize novel ‘reversed’ analytical sensors capable of generating an enhanced light signal when exposed to harsh environments, with potential impact in aerospace planning, environmental sensing and smart building.

## Methods

### Synthesis

Core-only CdSe CQWs were synthesized according to the procedure described in ref. 3. For the synthesis on CdSe/CdS core/shell CQW^22^, the first sulfur layer has been obtain by phase transfer of 5 ml of CdSe CQWs dispersed in hexane into 5 ml of N-methylformamide (NMF), to which 100 μl of ammonium sulfide (40%) was added. The reaction takes place in seconds and the polar solution was isolated. A mixture of acetonitrile and toluene (ratio of 20:80) was introduced to precipitate the CQWs and redissolve them in 3 ml of NMF. This was repeated at least two times in order to remove the sulfur precursor from the solution. The cadmium layer was formed by the addition of 5 ml of cadmium acetate in NMF (concentration of 0.5 M) and was rigorously stirred for 1 h. The CQWs were again purified as described above. For thicker shells, the same procedure was repeated until we reached the desirable thickness. The final CQWs were dispersed in 3 ml of toluene with the addition of 250 μl of oleylamine and 250 μl of oleic acid to stabilize them in solution.

### Spectroscopic studies

Absorption spectra of CQWs in solution were measured with a Varian Cary 50 spectrophotometer. Steady-state PL measurements were performed by exciting samples at 3.1 eV with *cw* diode lasers. The emitted light was dispersed with a spectrometer and detected with a charged-coupled device. The same setup coupled to an integrating sphere was used for PL quantum efficiency measurements on film samples. Transient PL measurements were carried out using frequency-doubled 250-fs pulses from a 78-MHz Ti:Sapphire laser at 3.1 eV. The emitted light was collected with a Hamamatsu streak camera (time resolution ~10 ps).

For oxygen pressure studies, a few monolayer films of CQWs were deposited on quartz substrates by dip casting from diluted hexane solutions (optical density of 0.07 at 500 nm; two dips for 10 s). The films were successively mounted in a sealed chamber with direct optical access, and the PL was monitored using the setup described above both as a function of the oxygen pressure and during O_2_/vacuum cycles to test the reversibility of the quenching process. Before any pressure ramp, the PL efficiency has been preliminary monitored for several minutes in O_2_ in order to evaluate possible photobrightening effects due to ultraviolet curing of surface defects. No photobrightening has been observed for any of the investigated samples. The same procedure has been followed for PL-sensing measurements using CO_2_, CO/argon (500 p.p.m. of CO) and humid air (20.5 g kg^−1^).

For magneto-optical measurements, the samples were mounted in the variable temperature insert of a split-coil cryo-magnet with direct optical access. The PL was excited with a continuous wave laser at 3.1 eV and the circularly polarized emission was selected using a quarter-wave plate coupled with a linear polarizer. The signal was revealed using a charge-coupled device camera coupled to a 6-m-long optical fibre to ensure full depolarization of the emitted light.

For single-particle PL-sensing experiments, the CQWs were deposited on a glass substrate from a very diluted hexane solution mounted in a Oxford Instrument Microscopy Cryostat. Fluorescence imaging was performed using a Nikon Ti-U inverted microscope and exciting the CQWs with a Xenon lamp filtered by a fluorescein isothiocyanate excitation filter (470–500-nm band-pass, excitation power density 500 mW cm^−2^, integration time 2 s).

Photographs of the CQW film and isolated particles in O_2_ and vacuum conditions were taken using a Canon EOS 400D camera.

### Spectroelectrochemical measurements

Indium-tin-oxide (ITO)-coated glass slides (50 × 7 × 0.7 mm, *R*_S_ <100 Ω) were purchased from Delta Technologies (part no. CG-90IN-CUV). The ITO-coated surface was first covered with zinc oxide (ZnO) nanoparticles (Nanograde, ~50 nm diameter) to avoid quenching of CQW emission by fast charge/energy transfer to ITO^54^,^55^. The ZnO NP layer (~60 nm thick, as measured using a Dektak profilometer) was deposited by dip coating the glass/ITO substrate into an ethanol suspension of ZnO nanoparticles (2 mg ml^−1^, one dip for 10 s) and annealed at 150 °C for 10 min in a nitrogen glovebox.

To test the stability of the glass/ITO/ZnO NP substrates during the potential scans, we performed control experiments in which we monitored changes in optical absorption spectra for prolonged exposures to negative and positive potentials^56^. The results of these measurements indicate that the substrates are unaffected by either positive or negative electrochemical potentials for exposure times of tens of minutes, which are much longer than the measurement time used in our SEC experiments (~100 s).

The CQWs were deposited onto the ZnO NP layer as a few-monolayer-thick film by dip coating from a dilute hexane solution (optical density of 0.07 at 500 nm; two dips for 10 s). The ITO was connected as a working electrode to the potentiostat (Bio Logic SP-200 Research Grade Potentiostat/Galvanostat) and the film was placed into a quartz cuvette filled with the electrolyte (0.1 M tetrabutylammonium perchlorate in propylene carbonate). Silver and platinum wires were used as quasi-reference and counter electrodes, respectively. All potentials reported in this work are measured relative to the quasi-reference silver electrode during staircase voltammetry scans (10 s scan rate).

The film was excited at 3.1 eV with continuous wave diode lasers and the emitted light was collected with a focusing lens and sent to a spectrometer coupled to a charge-coupled device array for spectral measurements.

## Author contributions

S.B. and F.M conceived the idea of reversed oxygen sensors using CQWs. S.C. and I.M. synthesized the materials and performed the TEM characterization. S.B., F.M. planned the experiments. M.L. performed the spectroelectrochemical experiments, the pressure studies and conducted the analysis of the results with F.M., I.M. and S.B. G.V. performed the magneto-PL experiments. J.P. carried out the single-particle sensing measurements. M.L. performed the calculations based on the model developed by S.B. and F.M.. S.B. wrote the paper in consultation with all of the authors.

## Additional information

**How to cite this article:** Lorenzon, M. *et al*. Reversed oxygen sensing using colloidal quantum wells towards highly emissive photoresponsive varnishes. *Nat. Commun*. 6:6434 doi: 10.1038/ncomms7434 (2015).

## Supplementary Material

Supplementary Information Supplementary Figures 1-15, Supplementary Tables 1-2 and Supplementary Discussion
