# Supplementary material for: Reversed oxygen sensing using colloidal quantum wells towards highly emissive photoresponsive varnishes
Source: Nat Commun. 2015 Mar 16;6:6434. doi: 10.1038/ncomms7434 (PMC4382706; doi:10.1038/ncomms7434)
Supplement: Supplementary Information — Supplementary Figures 1-15, Supplementary Tables 1-2 and Supplementary Discussion [file ncomms7434-s1.pdf]

Supplementary Fig. 1: Structural and optical properties of CdSe/1CdS CQWs and oxygen sensing ramp

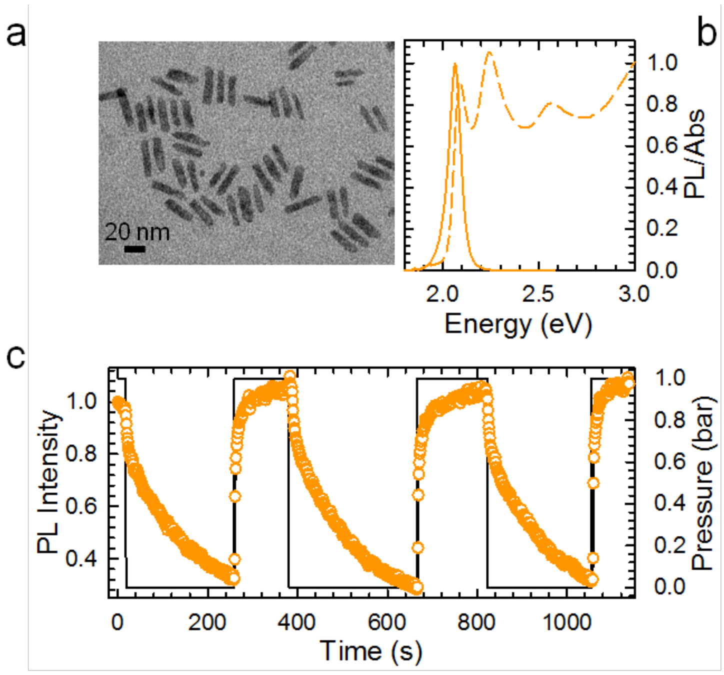

Supplementary Fig. 1. Structural and optical properties of CdSe/1CdS CQWs and oxygen sensing ramp. (a) Transmission electron micrographs of core/shell CdSe/1CdS CQWs, consisting of 2 nm thick CdSe core over-coated with 1 monolayer of CdS on each side (h=0.32 nm). (b) Optical absorption (dashed line) and photoluminescence (solid line) spectra of an hexane solution of core/shell CQWs under 3.1 eV excitation. (c) Integrated PL intensity of CdSe/1CdS CQWs during 'ON/OFF' O_2/vacuum cycles, starting from atmospheric pressure (1 bar) down to 10^{-4} bar. The pressure is shown as a black solid line. The PL intensity shows a ~65% dimming with respect to the initial values in O_2 and fully recovers after each cycle. Three cycles are reported to highlight the repeatability of the observed trend.

Supplementary Fig. 2: Photoluminescence spectra of CdSe and CdSe/CdS CQWs at 1bar and 10^{-4} bar O_2 pressure.

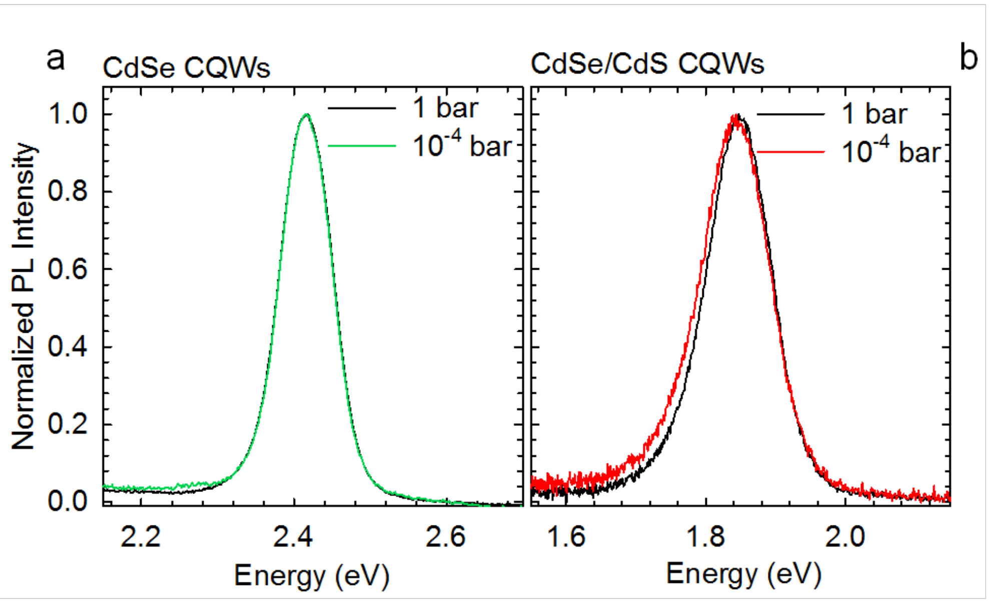

Supplementary Fig. 2 Photoluminescence spectra of CdSe and CdSe/CdS CQWs at 1bar and 10^{-4} bar O_2 pressure. Normalized PL spectra of (a) core-only (b) and core-shell CQWs (shell thickness h=0.95 nm) at 1 bar (black solid line) and 10^{-4} bar (green and red line, respectively). No significant shift of the PL spectra is observed, which indicates that the observed trends are essentially due to activation/passivation of surface traps and not to oxidation/reduction of the CQWs surface. All spectra are measured at room temperature under 3.1 eV excitation.

Supplementary Fig.3: Extended O_2 sensing cycling using CdSe CQWs

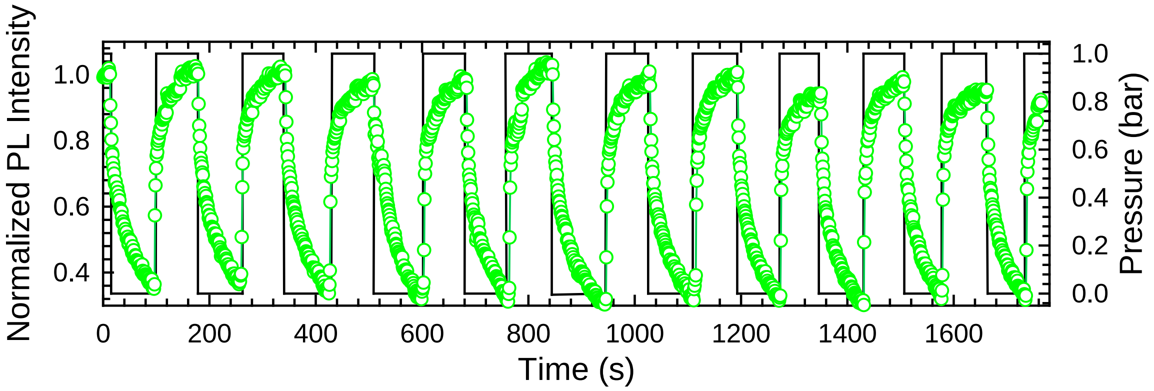

Supplementary Fig.3. Extended O_2 sensing cycling using CdSe CQWs. Integrated PL intensity of CdSe CQWs during 'ON/OFF' O_2/vacuum cycles starting from atmospheric pressure (1 bar) down to 10^{-4} bar. The pressure during the scan is shown as a black line. All measurements are performed at room temperature using 3.1 eV excitation. The data show full repeatability of the sensing response and complete recovery of the initial PL intensity for over ten consecutive O_2/vacuum ramps.

Supplementary Fig.4: Batch-to-batch reproducibility of the O_2 sensing ability of CdSe and CdSe/CdS CQWs

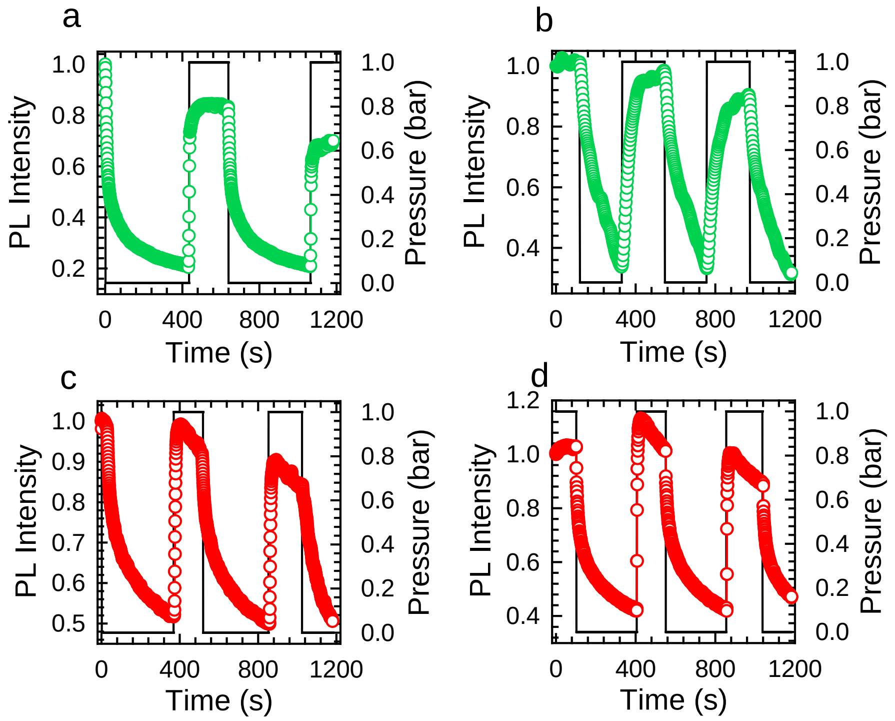

Supplementary Fig.4. Reproducibility of the O_2 sensing response for different batches of CdSe and CdSe/CdS core/shell CQWs. Integrated PL intensity of CdSe CQWs during 'ON/OFF' O_2/vacuum cycles, starting from 1 bar down to 10^{-4} bar. The pressure during the scan is shown as a black line. Data in (a) refer to a new film cast from the same batch studied in the main text that was synthesized about one year ago. The CdSe CQWs shown in (b) are from a new batch now synthesized solely to check the batch-to-batch reproducibility of the sensing response. Integrated PL intensity of CdSe/CdS CQWs (h=0.95 nm) during 'ON/OFF' O_2/vacuum cycles starting from atmospheric pressure (1 bar) down to 10^{-4} bar. Data in (c) refer to a new film cast from the same batch studied in the main text that was synthesized about one year ago. The CdSe CQWs shown in (d) are from a new batch synthesized now solely to check the batch-to-batch reproducibility of the sensing response. The results show the reproducibility of the sensing behavior of different batches, which is also well maintained over shelf time up to one year after the synthesis. All measurements are performed at room temperature using 3.1 eV excitation.

Supplementary Fig.5: Effect of illumination on the sensing response of CdSe CQWs

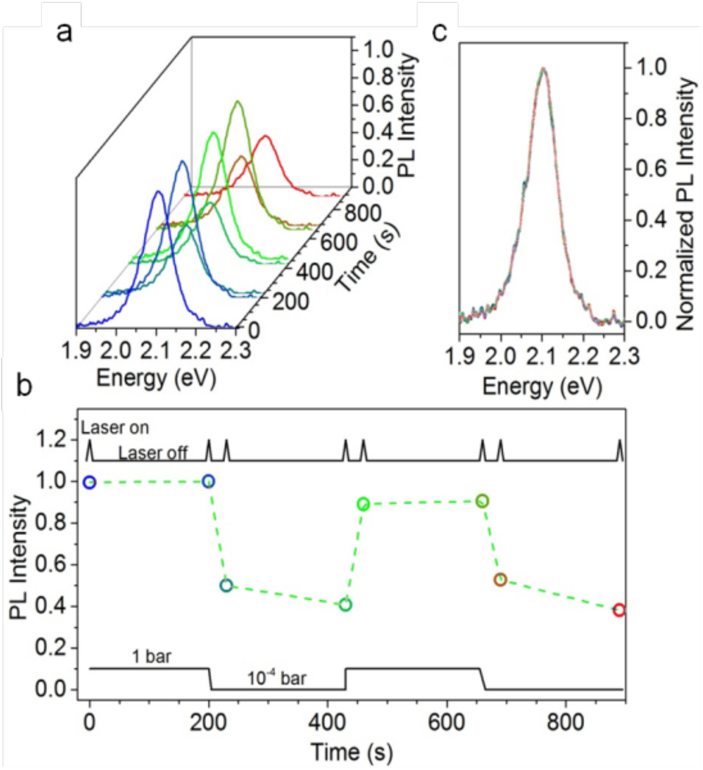

Supplementary Fig.5. Effect of illumination on the sensing response of CdSe CQWs. (a) Photoluminescence spectra and (b) integrated PL intensity of CdSe CQWs during ON/OFF O_2/vacuum cycles. The sample is kept in darkness except during the measurements of the emission spectrum when it is briefly illuminated (accumulation time 1 second). The PL is recorded in key conditions: in oxygen, just after evacuation, after 200 second at 10^{-4} bar, immediately after refilling with O_2 and after 200 s at 1 bar O_2 pressure. The scan is repeated twice to check the reproducibility of the response. The results show a similar behaviour to that observed under continuous illuminations, as shown both in Fig.2c in the main text and in Supplementary Fig.4. This indicates that illumination does not significantly affect the quenching dynamics. (c) Normalized PL spectra shown in ‘a’. No shift of the PL is observed in the different pressure conditions. The same colour scheme applies throughout the figure.

Supplementary Fig.6: Sensing response of CdSe CQWs to carbon monoxide and carbon dioxide

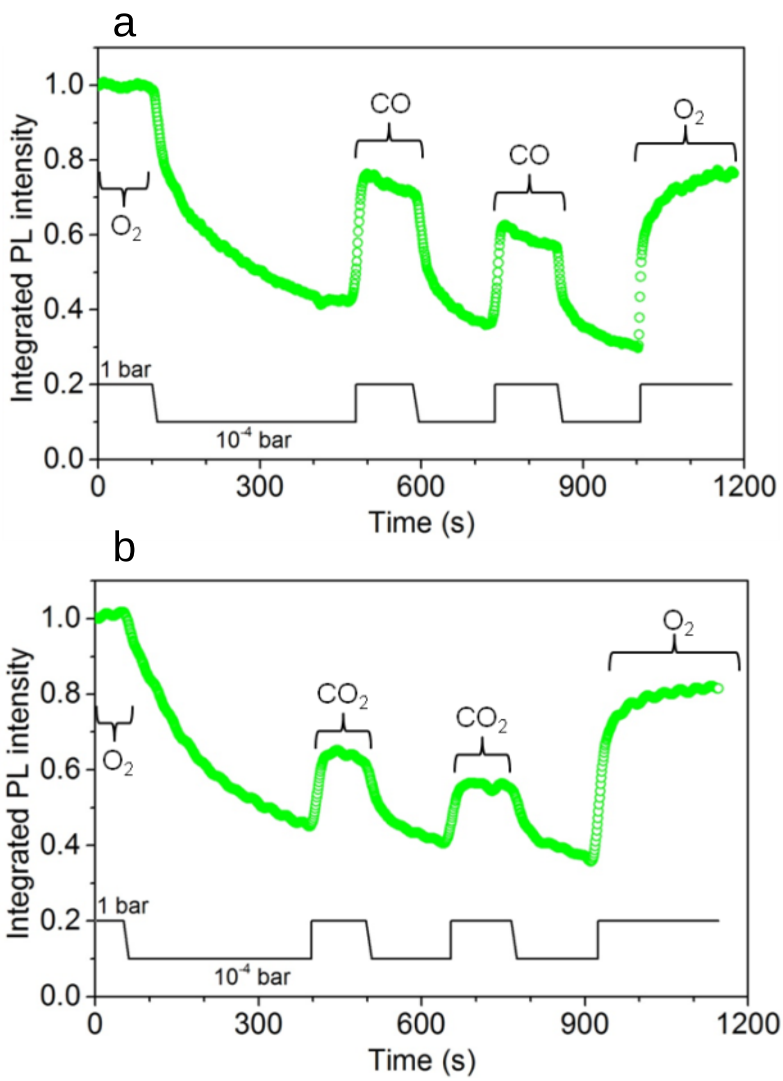

Supplementary Fig.6. Sensing response of CdSe CQWs to carbon monoxide and carbon dioxide. (a) Integrated PL intensity of CdSe CQWs during 'ON/OFF' gas/vacuum cycles. The scan starts from 1 bar in O_2 from where the chamber is evacuated to 10^{-4} bar. After about 400 seconds, the chamber is filled with a CO/Ar mixture (500 ppm of CO in argon) (1 bar). After about 100 seconds the chamber is evacuated again to 10^{-4} bar. The scan is repeated twice to check the reproducibility of the response, after which the chamber is filled with O_2 (1 bar). The data show the dimming of the PL upon lowering the chamber pressure. Interestingly, the presence of CO leads to partial recovery of the PL intensity indicating weak sensitivity of the CQWs to the gas. The PL intensity is almost fully recovered once the chamber is refilled with O_2. (b) Same experiment as in 'a' but using CO_2 gas. The pressure during the scan in both panels is shown as a black line. All measurements are performed at room temperature under 3.1 eV excitation.

Supplementary Fig.7: Effect of humidity on the sensing response of CdSe CQWs to oxygen

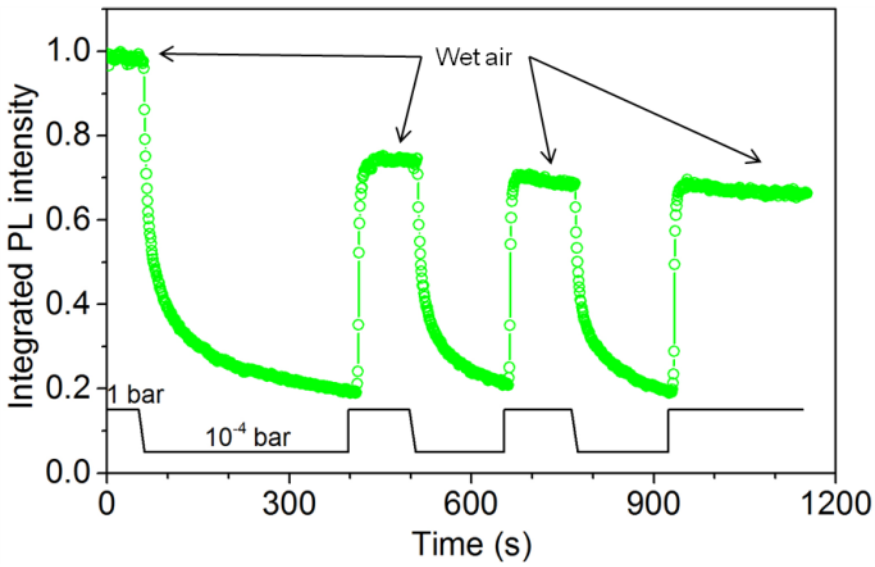

Supplementary Fig.7. Effect of humidity on the sensing response of CdSe CQWs to oxygen. Integrated PL intensity of CdSe CQWs during 'ON/OFF' air/vacuum cycles starting from atmospheric pressure (1 bar) down to 10^{-4} bar. The absolute humidity is 20.5 g/kg, corresponding to common ambient conditions in which sensing varnishes could be employed (i.e. about 85% humidity at room temperature). The pressure during the scan is shown as a black line. The measurement was performed at room temperature using 3.1 eV excitation. The data show repeatability of the sensing response with a weak effect of humidity on the recovery of the initial PL intensity.

Supplementary Fig.8: Spectrally resolved PL decay of CdSe CQWs during O_2/vacuum cycles.

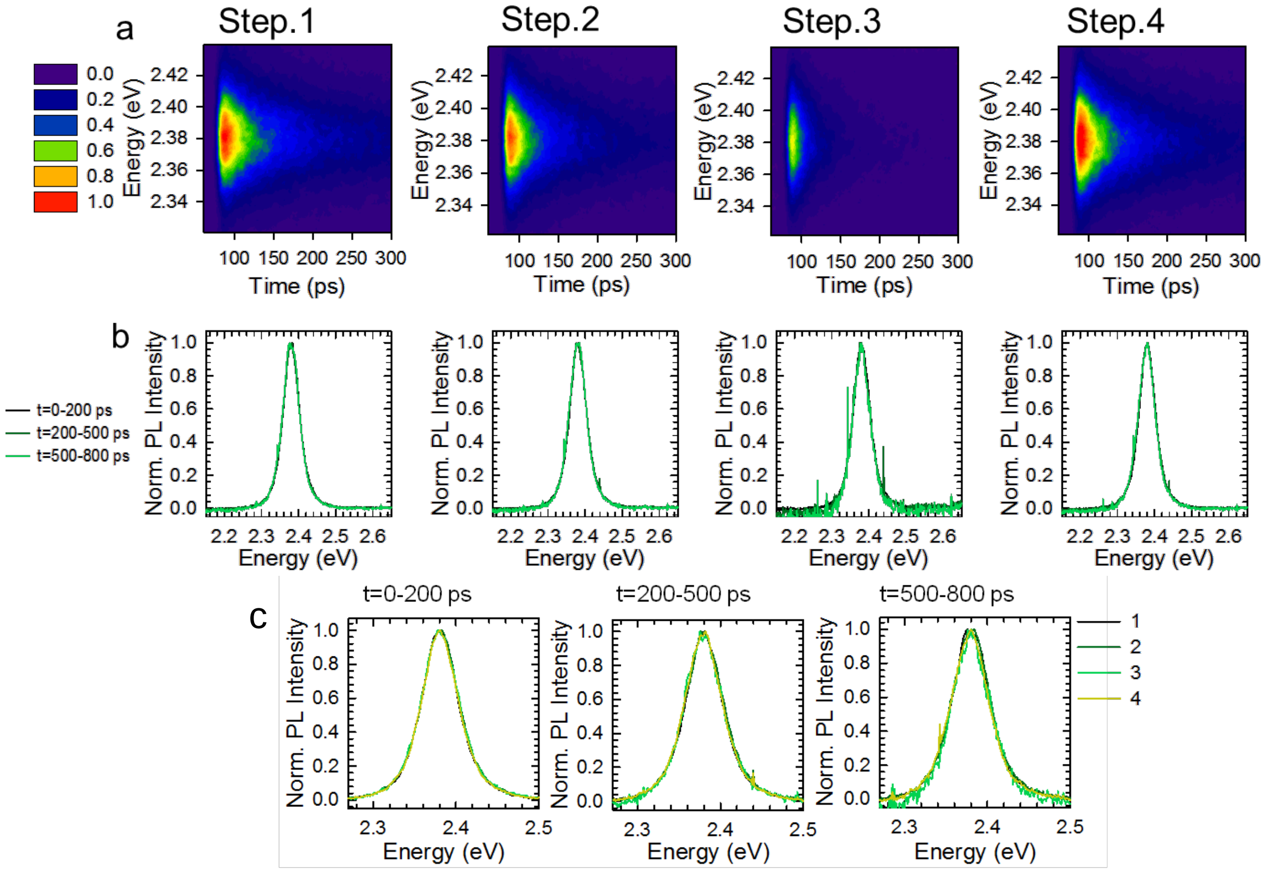

Supplementary Fig.8. Spectrally resolved PL decay of CdSe CQWs during O_2/vacuum cycles. (a) Contour plots of the spectrally resolved PL decay for CdSe CQWs. The panels correspond to the four steps of the O_2/vacuum cycles of Fig. 2c of the main text, namely: Step **1**, the initial condition of atmospheric pressure; Step **2**, the stage at which the chamber pressure is decreasing to ~10^{-4} bar; Step **3**, the end of the asymptotic PL dimming and Step **4**, the maximum of the PL signal after recovery. The images highlight the concomitant variations of the zero delay PL intensity ($I_{PL}^{t=0}$) and of the decay dynamics, as shown in Fig. 2d in the main text. (b) Normalized PL spectra of core-only CQWs at different delay time after the excitation pulse (t=0-200 ps, t=200-500 ps, t=500-800 ps) extracted from the contour plots in ‘a’. (c) Time resolved PL spectra at increasing time from the excitation pulse, namely, 0-200 ps, 200-500 ps and 500-800 ps, extracted from the contour plots in ‘a’. No significant shift is observed at different pressure values nor during the emission decay. All measurements are performed at room temperature using 3.1 eV excitation.

Supplementary Fig.9: Spectrally resolved decay of CdSe/CdS CQWs during O_2/vacuum cycles

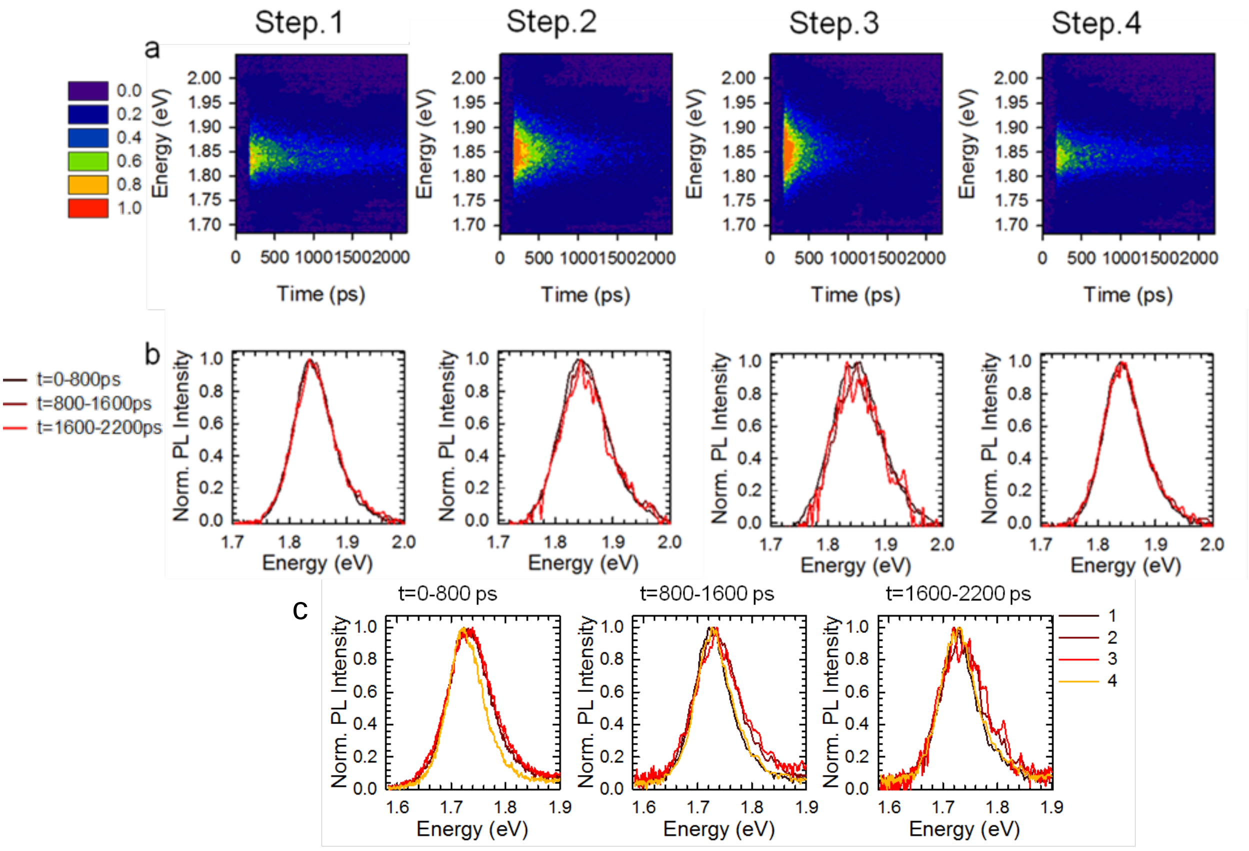

Supplementary Fig.9. Spectrally resolved PL decay of CdSe/CdS CQWs during O_2/vacuum cycles. (a) Contour plots of the spectrally resolved PL decay for CdSe/CdS core/shell CQWs (shell thickness h=0.95 nm). The panels correspond to the four steps of the O_2/vacuum cycles of Fig. 2c of the main text, namely: Step **1**, the initial condition of atmospheric pressure; Step **2**, the stage at which the chamber pressure is decreasing to ~10^{-4} bar; Step **3**, the end of the asymptotic PL dimming and Step **4**, the maximum of the PL signal after recovery. The images highlight the concomitant variations of the zero delay PL intensity ($I_{PL}^{t=0}$) and of the decay dynamics, as shown in Fig. 2e in the main text. (b) Normalized PL spectra of core/shell CQWs at different delay time after the excitation pulse (t=0-800 ps, t=800-1600 ps, t=1600-2200 ps) extracted from the contour plots in ‘a’. (c) Time resolved PL spectra at increasing time from the excitation pulse, namely 0-800 ps, 800-1600 ps and 1600-2200 ps. No significant shift is observed at different pressure values nor during the emission decay. All measurements are performed at room temperature using 3.1 eV excitation.

Supplementary Fig.10: Double-exponential fits of the PL decay curves

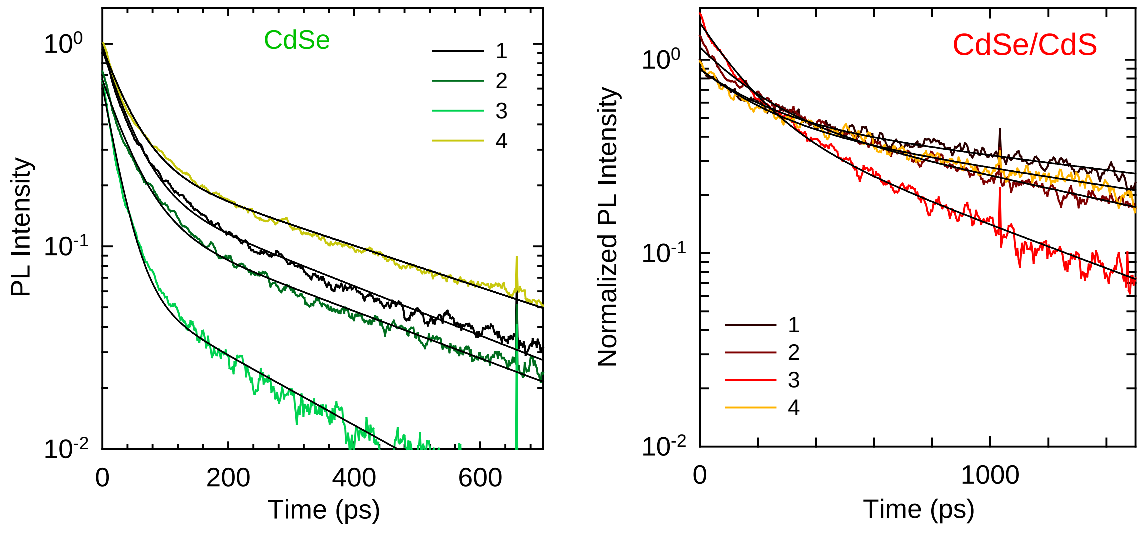

Supplementary Fig.10. Double-exponential fits of the PL decay curves. PL decay curves of core only and core/shell CdSe/CdS CQWs measured at the respective steps of the pressure/vacuum cycles in Fig.2b. The fits to double exponential functions are reported as black lines.

Supplementary Fig. 11: Fast decay rate and initial PL intensity of CdSe and CdSe/CdS CQWs at the different steps of the O_2/vacuum cycle in main Fig.2c

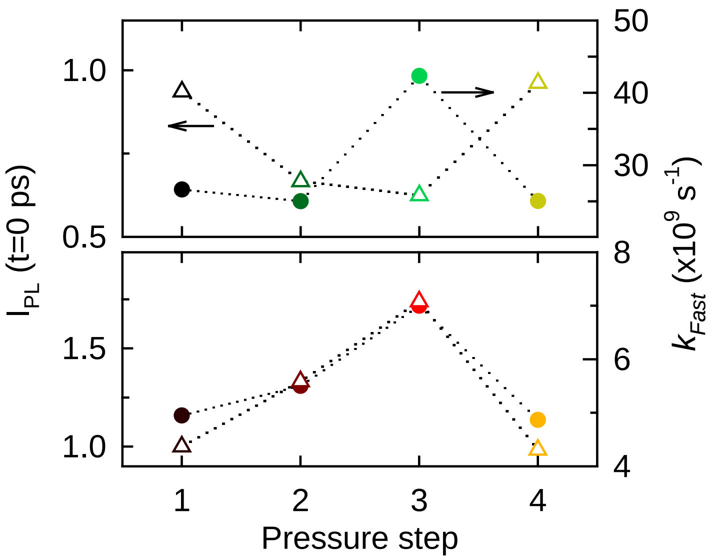

Supplementary Fig.11. Fast decay rate and initial PL intensity of CdSe and CdSe/CdS CQWs at the different steps of the O_2/vacuum cycle in main Fig.2c. Decay rate of the fast component of the bi-exponential dynamics (triangles) and initial PL intensity, I_{PL}(t=0ps) (triangles) for both core only (top panel) and core/shell (lower panel) CQWs. All measurements are performed at room temperature using 3.1 eV excitation.

Supplementary Fig.12. Single particle investigation of the sensing response of CdSe CQWs

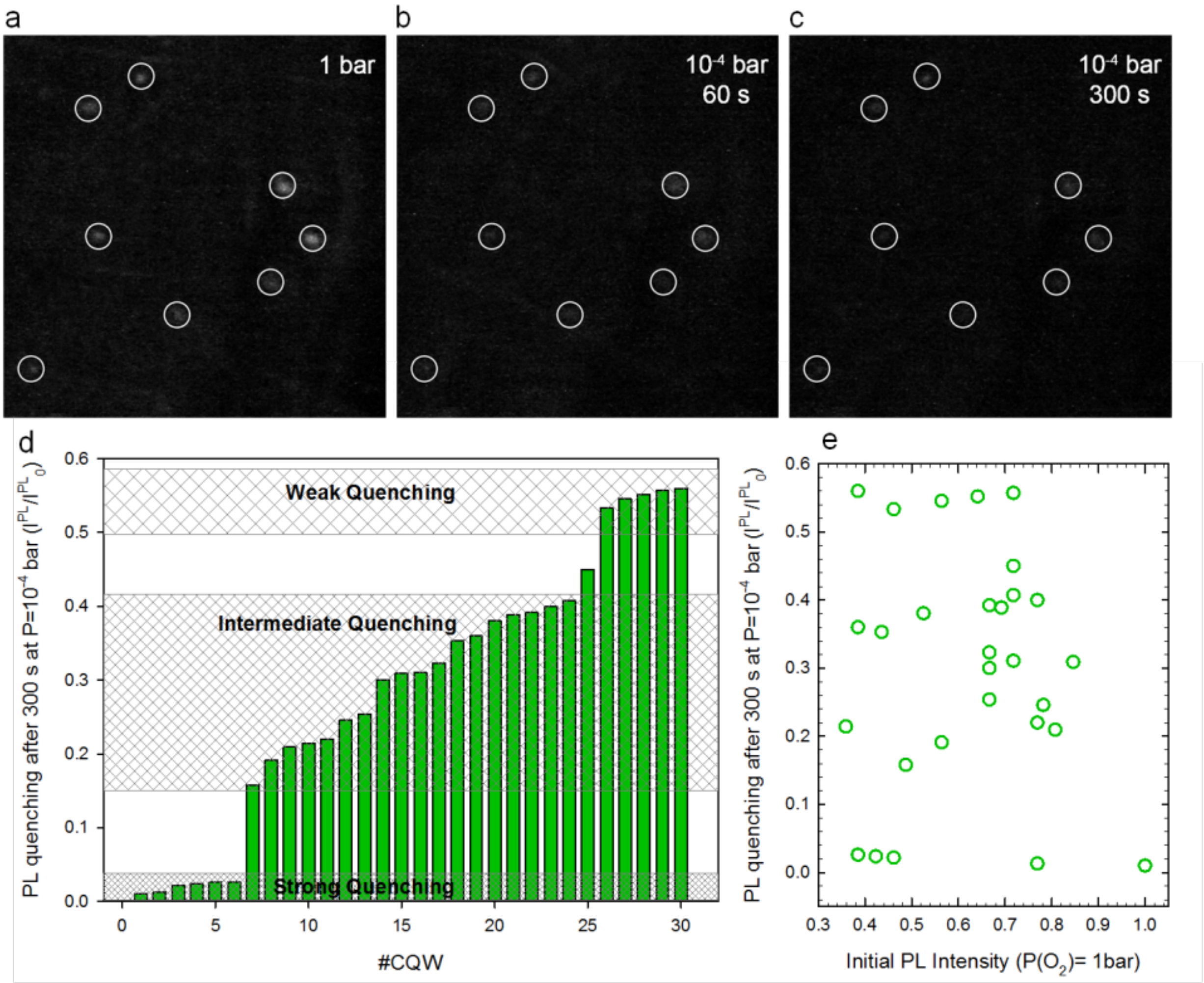

Supplementary Fig.12. Single particle investigation of the sensing response of CdSe CQWs. Photographs of representative individual CQWs at (a) 1 bar oxygen pressure, (b) 60 seconds and (c) 300 seconds after evacuation at 10^{-4} bar, showing the progressive dimming of the emission intensity upon lowering the chamber pressure and prolonged exposure to vacuum. Individual particles are highlighted with circles. (d) PL quenching as defined as the ratio between the initial PL intensity and the intensity after 300 seconds at 10^{-4} bar for 30 individual CQWs. The histogram highlights three subpopulations showing strong, intermediate and weak quenching upon lowering the O_2 pressure. Importantly, the sensing response is independent on the initial PL intensity, as shown in the correlation plot of the PL quenching vs. initial PL intensity in (e).

Supplementary Fig.13: Circular polarization-resolved PL spectra of CdSe CQWs

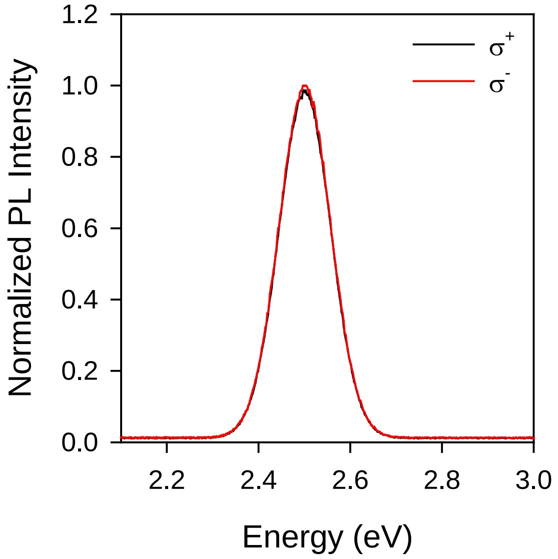

Supplementary Fig.13. Circular polarization-resolved PL spectra of CdSe CQWs. Circular polarization-resolved PL spectra of CdSe CQWs at magnetic field of B=5 T and T=2.5K measured using 3.1 eV excitation. The clockwise (σ^+) and counter-clockwise (σ^-) emission spectra are essentially identical and are reported in black and red lines, respectively.

Supplementary Fig.14: Spectro-electrochemical measurements of CdSe and CdSe/CdS CQWs as a function of negative electrochemical potential

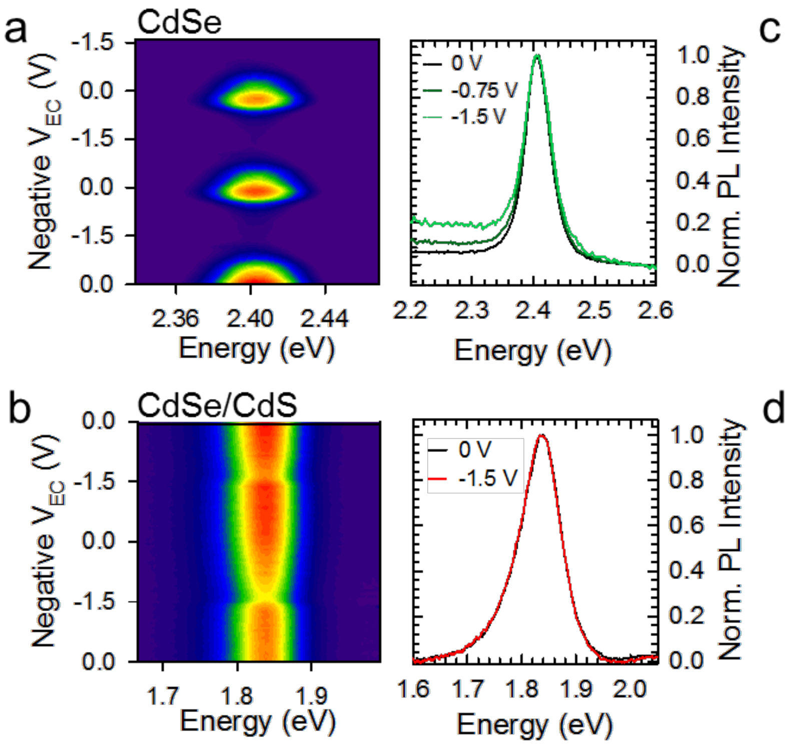

Supplementary Fig.14. Contour plots of the spectrally resolved PL intensity of (a) CdSe and (b) CdSe/CdS (shell thickness h=0.95 nm) CQWs as a function of negative EC potential from 0 V to -1.5 V. Consecutive stepwise scans are reported to highlight the repeatability of the process. Normalized PL spectra at (c) 0 V, -0.75 V and -1.5 V for CdSe CQWs (extracted from the data in 'a') and (d) 0 V and -1.5 V for CdSe/CdS CQWs (extracted from the data in 'b'). No shift of the PL spectra is observed during the SEC scan, indicating that the PL intensity trends are due to activation/passivation of surface traps and not to oxidation/reduction of the CQWs surfaces.

Supplementary Fig.15: Spectro-electrochemical measurements on CdSe and CdSe/CdS CQWs as a function of positive electrochemical potential

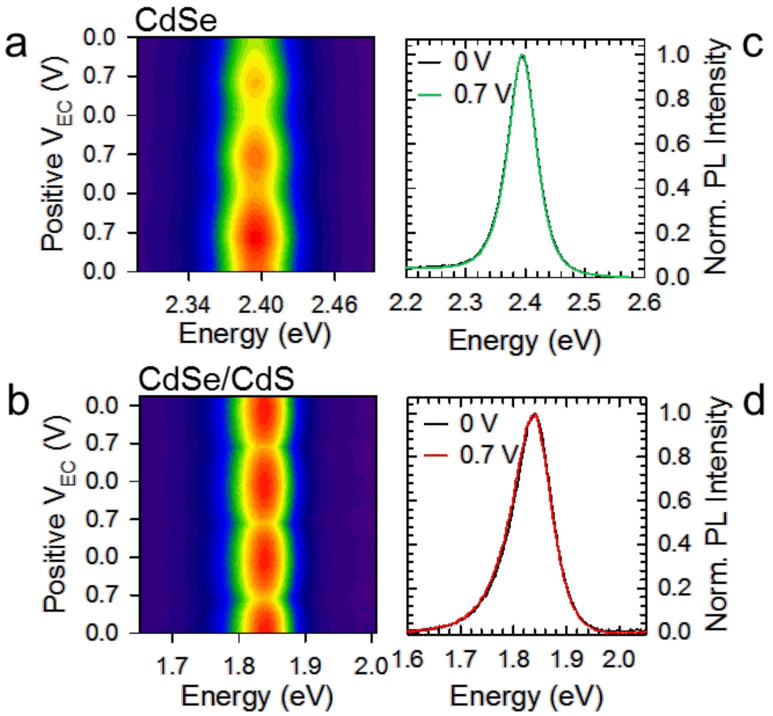

Supplementary Fig.15. Contour plots of the spectrally resolved PL intensity of (a) CdSe and (b) CdSe/CdS (shell thickness h=0.95 nm) CQWs as a function of positive EC potential from 0 V to +0.7 V. Consecutive stepwise scans are reported to highlight the repeatability of the process. Normalized PL spectra at (c) 0 V and +0.7 V for CdSe CQWs (extracted from the data in 'a') and (d) 0 V and +0.7 V for CdSe/CdS CQWs (extracted from the data in 'b'). No shift of the PL spectra is observed during the SEC scan, indicating that the PL intensity trends are due to activation/passivation of surface traps and not to oxidation/reduction of the CQWs surfaces.

Supplementary Table 1. Fitting parameters

**CdSe CQWs**	I_0	I_F	k_F (ns^{-1})	I_S	k_S (ns^{-1})	$A_F=I_F/k_F$	$A_S=I_S/k_S$	$W_F=A_F/(A_F+A_S)$	$W_S=A_S/(A_F+A_S)$
Step 1	0.94	0.74	26.6	0.20	2.80	0.03	0.07	0.29	0.71
Step 2	0.67	0.53	25.0	0.14	2.68	0.02	0.05	0.29	0.71
Step 3	0.62	0.56	42.3	0.06	3.95	0.01	0.02	0.45	0.55
Step 4	0.96	0.70	25.0	0.26	2.36	0.03	0.11	0.20	0.80
**CdSe/CdS CQWs**	I_0	I_F	k_F (ns^{-1})	I_S	k_S (ns^{-1})	$A_F=I_F/k_F$	$A_S=I_S/k_S$	$W_F=A_F/(A_F+A_S)$	$W_S=A_S/(A_F+A_S)$
Step 1	0.90	0.41	4.95	0.49	0.42	0.08	1.16	0.07	0.93
Step 2	1.17	0.64	5.50	0.52	0.73	0.12	0.71	0.14	0.86
Step 3	1.56	1.06	7.00	0.508	1.29	0.15	0.39	0.28	0.72
Step 4	0.90	0.44	4.87	0.46	0.52	0.09	0.89	0.09	0.91

The decays are fitted with a biexponential function, from which we deduce the amplitudes *Ii* (not listed), decay rates k_i, and corresponding areas $A_i = I_i/k_i$, and relative weights $W_i = A_i / (A_1+A_2)$. For both core-only and core/shell CQWs, the radiative decay dominates in O_2 atmosphere, with weights of 72-80% and 91-93%, respectively (steps 1 and 4).

In both samples, the total area *A1+A2* (proportional to the number of photons emitted and thus the overall quantum efficiency) decreases in steps 2-3, indicating more efficient carrier trapping under vacuum, yet for core/shell CQWs the area of fastest component increases. This suggests a competing mechanism that enhances the efficiency of this decay channel, *in casu* the formation of trions that have a faster recombination rate.

Supplementary Table 2: Model parameters

	CdSe CQWs	CdSe/CdS CQWs
k_{rad}	3 ns^{-1}	0.4 ns^{-1}
k_{ET}	0.2 ns^{-1}	0.5 ns^{-1}
k_{HT}	30 ns^{-1}	0.3 ns^{-1}
k_{DT}	0.2 ns^{-1}	0.2 ns^{-1}
$\tilde{N}_0$	0.15 V	0.01V
$\tilde{n}_0$	0.05 V	0.05 V
k	10 V^{-1}	10 V^{-1}

Supplementary Discussion

Here, we report the rate equations that describe the model depicted in Fig. 4 (main text).
We denote the populations of electrons in the conduction band as *n*, holes in the valence band as *p*, and trapped electron (holes) at defect sites as n_T (p_T). The excitation rate is kept constant to an arbitrary value. To express the variation of trap population with the EC potential, V_{EC}, we introduce a proportionality constant k ([1/V]) which allows us to express the relative trap occupancies in the units of voltage. We thus describe the number of empty electron traps as $\tilde{N}$ - $\tilde{n}_0+V_{EC}$, where $\tilde{N}_0$ = N_0/k is the effective width of the defect band and N_0 is the number of traps per individual NS. $\tilde{n}_0$ = n_0/k is the initial occupancy of the defect band at V_{EC} = 0 V, and n_0 is the number of occupied traps per individual NS in the absence of photoexcitation. The positive sign of V_{EC} accounts for the fact that negative EC potentials raise the Fermi level thereby reducing the number of empty electron traps. Similarly, we describe the number of active hole traps as $\tilde{n}_0$-V_{EC}.
For both systems, we consider exclusively the long-lived portion of the CQW ensemble as it is responsible for the majority of the luminescence signal. The detrapping rate k_{DT} has been chosen slow enough so as to not interfere with the charge extraction processes yet non-zero so as to describe a closed system. This is in agreement with recent observations that detrapping in spherical nanocrystals can take up to several minutes[56]. For core-only CQWs, the hole trapping rate has been chosen to be much larger than the radiative rate, in agreement with the ultrafast ‘static’ quenching observed in the time resolved PL measurements. Electron trapping rate is instead negligible. The rates for the core/shell CQWs reflect the balance between the radiative recombination channel and the competitive nonradiative relaxation pathways that indeed lead to symmetric quenching effect under positive and negative electrochemical potentials.
Once the steady state populations are obtained, we calculate the dependences the emission intensity on V_{EC} from $k_{rad} \cdot n \cdot p$ and show them in Fig. 4d,e as a function of the EC potential and compute the PL intensity as $I_{PL}=n*p*k_{rad}$.

Within this assumption, we can write the following rate equations for *n*, *p*, n_T and p_T as a function of the EV potential:

$$dn/dt= k_{ex} - k_{rad} \cdot p \cdot n - k_{ET} \cdot n \cdot (\tilde{N}_0 - \tilde{n}_0 + V_{EC} + p_T) - k_{DT} \cdot n \cdot p_T ; \qquad (1)$$

$$dp/dt = k_{ex} - k_{rad} \cdot p \cdot n - k_{HT} \cdot p \cdot (\tilde{n}_0 - V_{EC} + n_T) - k_{DT} \cdot n_T \cdot p ; \qquad (2)$$

$$dn_T/dt = k_{ET} \cdot n \cdot (\tilde{N}_0 - \tilde{n}_0 + V_{EC} + p_T) - k_{DT} \cdot n_T \cdot p ; \qquad (3)$$

$$dp_T/dt = k_{HT} \cdot p \cdot (\tilde{n}_0 - V_{EC} + n_T) - k_{DT} \cdot n \cdot p_T ; \qquad (4)$$

where the occupancies of the electron and hole states are related by the condition of charge neutrality:

$$n + n_T = p + p_T.$$

The numerical solution of the system of the rate equations is performed in the steady state regime (time derivatives equal to zero) using the values for the rates inferred from the measurements (see Supplementary Table 2) or, when not directly accessible, chosen accordingly to the observed phenomenology in order to qualitatively reproduce the main experimental trends.
